# Changes in clinical and laboratory features of Kawasaki disease noted over time in Daejeon, Korea

**DOI:** 10.1186/s12969-017-0192-y

**Published:** 2017-08-07

**Authors:** Hong-Ryang Kil, Jae-Won Yu, Sung-Churl Lee, Jung-Woo Rhim, Kyung-Yil Lee

**Affiliations:** 1 Department of Pediatrics, Chungnam National University Hospital, School of Medicine, Chungnam National University, Daejeon, Korea; 2 Departments of Pediatrics, Daejeon St. Mary’s Hospital, College of Medicine, The Catholic University of Korea, 64 Daeheung-ro, Jung-gu, Daejeon, 34943 Republic of Korea

**Keywords:** Kawasaki disease, Epidemiology, Clinical signs, coronary artery lesions

## Abstract

**Background:**

Kawasaki disease (KD) becomes one of the common diseases in Korea. Changes in clinical features and laboratory findings of KD were evaluated over a period of 10 years.

**Methods:**

We reviewed the medical records of KD patients and compared the clinical and laboratory features of two KD patient groups: those admitted from 2000 to 2004 (group A, 284 cases) and those admitted from 2010 to 2014 (group B, 331 cases).

**Results:**

There were a total of 615 KD patients (mean age: 29.7 months; male-to-female ratio = 1.6:1), including 228 incomplete KD patients. Incomplete KD patients had milder values in some laboratory indices. The preadmission and total fever durations were longer in group A than in group B. The proportion of incomplete KD was higher in group B, but incidence of coronary artery lesions (CALs) was lower. For laboratory indices, the C-reactive protein and follow-up platelet values were lower, and the hemoglobin and albumin values were higher in group B. The same clinical and laboratory findings were confirmed in the KD subgroups; those with the same fever duration of 5 or 6 days and same ages, those with complete KD, and those with incomplete KD in the two different time periods.

**Conclusions:**

Our findings suggest that clinical features of KD tend to be milder over time and manifest in a higher incidence of incomplete KD, lower incidence of CALs, and less severe laboratory findings in recent KD patients in Korea compared with their historic counterparts.

## Background

Kawasaki disease (KD) is an acute self-limiting systemic inflammation that can involve multiple organs, especially the heart via coronary artery lesions (CALs) [1]. KD is a newly appeared disease in the East Asian countries, including Japan, South Korea, Taiwan and China; though infantile polyarteritis nodosa, which may be a severe form of KD, has been reported in the Western countries since the late nineteenth century [2]. KD began being reported in Asian countries in specific time periods: in the early 1960s in Japan, 1970s in Korea and in 1970–80s in Taiwan and China, and possibly 1990s in India and other economically grown Asian countries [3–7], which suggests that economic growth and westernization of these countries are associated with the emergence of KD [8, 9]. KD has shown epidemiological characteristics in these countries; it has the same age predilection of 6 months-4 years and has become a nationwide endemic disease within 2 decades with the incidence slowly increasing after its first report. Additionally, KD occurs throughout the year with slight monthly and regional variations. On the other hand, the incidence of KD in Western countries is lower by tenth to one twentieth to Asian countries and has plateaued during recent decades [10]. These unique epidemiological characteristics are not observed in any newly emerging infectious diseases, such as acquired immune deficiency syndrome (AIDS) and severe acute respiratory syndrome (SARS). Thus, it is proposed that KD may be an acute immune-mediated disease that develops after an infection with an unknown pathogen(s) in genetically susceptible immune-immature young children, and that the immunopathogenesis of KD may be similar to that of infection-related immune-mediated diseases such as acute rheumatic fever rather than that of infectious disease such as scarlet fever [9].

It has been reported that epidemiological and/or clinical characteristics of infectious diseases, including scarlet fever, and infection-related immune-mediated diseases, including acute rheumatic fever and acute post-infectious glomerulonephritis (APSGN), have changed over time [11–13]. Nationwide epidemiological studies in Japan and Korea have reported that the epidemiology of KD has also changed over time, including increased incomplete KD and less severe CALs in recent patients compared to past KD patients [14, 15]. We also independently observed similar results in neighboring hospitals in Daejeon, Korea, which suggests that KD in recent patients has a milder phenotype compared to that in past KD patients [16, 17].

In this study, we evaluated clinical and laboratory findings of Korean KD patients to determine if there were changes in recent KD patients compared to past KD patients. We also discussed clinical implications of changing KD epidemiology in Korea.

## Methods

This study was conducted in two university hospitals in Daejeon, Korea: Chungnam National University Hospital (CNUH) and The Catholic University of Korea Daejeon St. Mary’s Hospital (DSMH). To evaluate the changes in clinical features more clearly, we divided the subjects into 2 groups according to their time of admission, a decade-interval apart.

We reviewed medical records of a total of 615 KD patients: those admitted between January 2000 and December 2004 (the 2000–2004 group, *n* = 284) and those admitted between January 2010 and December 2014 (the 2010–2014 group, *n* = 331). The selection of KD was based on clinical diagnostic criteria guidelines established by the Japanese Kawasaki Disease Research Committee or the American Heart Association (AHA) [1, 18]. There were 330 cases from CNUH and 285 cases from DSMH. KD patients that had spontaneous defervescence without IVIG treatment during hospitalization were excluded (28 cases).

Complete KD is defined as having fever of ≥5 days with at least 4 of the 5 principal clinical signs: 1) bilateral conjunctival injections, 2) changes in the lips and oral cavity, 3) polymorphous exanthema, 4) changes in the peripheral extremities, and 5) acute nonpurulent cervical lymphadenopathy. Incomplete KD was defined as having fever of ≥5 days with 3 or fewer principal signs, with or without cardiac lesions once other KD-like diseases with similar findings had been excluded. Because the diagnosis of KD in this study was made by experts in KD, independently, at each hospital during the study period (HR Kil and KY Lee), there were few differences in the policy of diagnosis and IVIG treatment in this study.

Laboratory parameters were examined at least twice during hospitalization at presentation and at discharge. All patients underwent 2D–echocardiography of the coronary arteries during hospitalization and again at approximately 1 or 2 months after discharge. Echocardiographic findings that were performed during hospitalization were analyzed. CALs were defined as ectasia when there was coronary arterial dilatation ≤4 mm in children that were younger than 5 years or ≤5 mm in children that were 5 years or older, or when the diameter was <1.5 times the size of the adjacent coronary artery. Aneurysm was defined as coronary artery dilatation in excess of that ascribed to ectasia, according to the Japanese Ministry of Health and Welfare guidelines [18]. We compared the clinical and laboratory indices between the groups.

### Statistical analysis

All calculations were performed using SPSS ver. 14.0 (SPSS Inc., Chicago, IL, USA). Continuous variables were expressed as mean ± standard deviation. Student’s t-test for continuous variables was used for comparisons between the groups. The Pearson’s Chi-square test was used for categorical variables. A *P* value less than 0.05 was considered statistically significant.

## Results

### Clinical characteristics of total KD patients

The mean age of all of the KD patients (*n* = 615) was 29.7 ± 21.3 months, and the male-to-female ratio was 1.6:1 (385:240). Among the total patients, 387 showed complete KD and 228 showed incomplete KD at presentation. The prevalence rates of clinical diagnostic signs of the eyes, lips, skin rashes, extremity changes, and cervical lymphadenopathy were 92.7%, 84.1%, 63.4%, 81%, and 51.7%, respectively. In complete KD patients (*n* = 387), the prevalence rates were 96.9%, 96.1%, 86.3%, 93.8%, and 63.0%, respectively, and 35.9% of the patients (139/387) had all 5 diagnostic signs. In incomplete KD patients (*n* = 228), 149 patients had 3 diagnostic signs, and 79 patients had ≤2 diagnostic signs (5 patients had one sign of lymphadenopathy). IVIG treatment for incomplete KD was based on laboratory indices; incomplete KD patients in the 2000–2004 group, before the 2004 AHA guidelines were published, had WBC count of >10,000/mm^3^, neutrophil differential of >50%, and CRP level of >2 mg/dL in the early stage of the illness with repeated examinations, and they showed an increased platelet count at follow-up examination after IVIG treatment [16, 19]. Diagnosis and IVIG treatment of incomplete KD patients in the 2010–2014 group were referred to the AHA guidelines [1]. All patients received IVIG (2 g/kg) for 10–12 h, and the mean duration from admission day to IVIG infusion day was 1.2 ± 0.9 days. A majority of KD patients (89.1%, 548/615) received IVIG within 48 h after admission, and there was no difference in time-gap between the 2000–2004 group and the 2010–2014 group (1.2 ± 1.0 days vs. 1.2 ± 0.9 days, *P* = 0.732). However, the incomplete KD group received IVIG later than the complete KD group (1.4 ± 2.1 days vs. 1.1 ± 0.9 days, *P* < 0.001).

### Comparison of clinical features between complete KD and incomplete KD

Epidemiological and clinical data of complete and incomplete KD groups are shown in Table 1. There were no significant differences in clinical parameters between the two groups based on age, sex, duration of fever, or proportion of CALs. The mean hospitalization time was longer (7.6 days vs. 6.6 days, *P* < 0.001) and the proportion of repeated IVIG-treated cases was higher in the complete KD cases (11.9% vs. 5.7%, *P* = 0.015). Laboratory parameter analysis indicated differences in some parameters, including neutrophil/lymphocyte differential, CRP (10 mg/dL vs. 8.8 mg/dL, *P* = 0.016), albumin (3.6 g/dL vs. 3.7 g/dL, *P* = 0.004), and ALT (127 IU/L vs. 95 IU/L, *P* = 0.018), between the groups. In ALT, 42.4% of complete KD and 53.9% of incomplete KD were not increased at presentation (*P* = 0.006), and 36.7% of complete KD and 24.1% of incomplete KD had values >100 IU/L (*P* = 0.001, Table 1).

**Table 1:** Clinical and laboratory findings in complete KD and incomplete KD

	Complete KD (*n* = 387)	Incomplete KD (*n* = 228)	*P*
Age (month)	30.9 ± 20.3	27.7 ± 22.9	0.069
Sex (male/female)	233/154	142/86	0.669
Fever (day)			
Preadmission	4.5 ± 1.7	4.6 ± 2.1	0.795
Total	6.8 ± 2.6	6.6 ± 2.7	0.515
Hospitalization (day)	7.6 ± 3.6	6.6 ± 3.2	< 0.001
Repeated IVIG, n (%)	46 (11.9)	13 (5.7)	0.015
CALs, n (%)	73 (18.9)	46 (20.2)	0.751
Ectasia	69 (17.8)	42 (18.4)	0.914
Aneurysm	4 (1.0)	4 (1.8)	0.477
WBC (/mm^3^)	15,000 ± 5100	15,000 ± 5000	0.991
Neutrophil (%)	66.1 ± 15.4	61.3 ± 15.6	< 0.001
Lymphocyte (%)	22.5 ± 12.4	26.8 ± 12.4	< 0.001
Hemoglobin (g/dL)	11.1 ± 1.1	11.2 ± 1.0	0.665
Platelet (x-10^3^/mm^3^)	323 ± 124	335 ± 140	0.086
Platelet, follow-up	473 ± 189	456 ± 174	0.098
CRP (mg/dL)	10.0 ± 6.3	8.8 ± 5.7	0.016
ESR (mm/h)	66 ± 31	67 ± 27	0.593
Total protein (g/dL)	6.5 ± 0.7	6.5 ± 0.6	0.995
Albumin (g/dL)	3.6 ± 0.4	3.7 ± 0.5	0.004
AST (IU/L)	96 ± 161	79 ± 241	0.304
ALT (IU/L)	127 ± 155	95 ± 171	0.018
ALT <40 IU, n (%)	164 (42.4)	123 (53.9)	0.006
ALT 41–99 IU, n (%)	81 (20.9)	50 (21.9)	0.761
ALT >100 IU, n (%)	142 (36.7)	55 (24.1)	0.001

Values are mean ± SD or n (%)

*KD* Kawasaki disease, *IVIG* intravenous immunoglobulin, *CALs* coronary artery lesions, *WBC* white blood cell, *CRP* C-reactive protein, *ESR* erythrocyte sedimentation rate, *AST* aspartate aminotransferase, *ALT* alanine aminotransferase

### Comparison of epidemiological and clinical features between the 2000–2004 group and the 2010–2014 group

Demographic, clinical, and laboratory parameters are shown in Table 2. The mean age and male-to-female ratio did not differ between the groups. In terms of clinical features, fever duration and hospitalization stay were shorter, while the proportion of incomplete KD was higher in the 2010–2014 group (26.4% vs. 46.2%, *P* < 0.001). The rates of repeated IVIG treatment were not significantly different between the groups. The rate of total CALs was lower (23.6% vs. 15.7%, *P* = 0.014) and severe CALs (aneurysm) tended to decrease in the 2010–2014 (2.1% vs. 0.6%) compared to the 2000–2004 group.

**Table 2:** Clinical and laboratory findings in the 2000–2004 group and the 2010–2014 group (*n* = 615)

	2000–2004 (*n* = 284)	2010–2014 (*n* = 331)	*P*
Age (month)	30.5 ± 22.3	29.1 ± 20.5	0.433
Sex (M/F)	168/116	207/124	0.408
Fever (day)			
Preadmission	4.9 ± 2.1	4.2 ± 1.5	< 0.001
Total	7.2 ± 3.2	6.4 ± 2.0	< 0.001
Hospitalization (d)	8.5 ± 4.1	6.1 ± 2.4	< 0.001
Incomplete KD, n (%)	75 (26.4)	153 (46.2)	< 0.001
Repeated IVIG, n (%)	26 (9.2)	33 (10)	0.784
CALs, n (%)	67 (23.6)	52 (15.7)	0.014
Ectasia	61 (21.5)	50 (15.1)	0.046
Aneurysm	6 (2.1)	2 (0.6)	0.153
WBC (/mm^3^)	15,200 ± 5100	14,900 ± 5200	0.476
Neutrophil (%)	63.4 ± 16.5	65 ± 14.8	0.226
Lymphocyte (%)	23.4 ± 13	24.7 ± 12.2	0.197
Hemoglobin (g/dL)	10.8 ± 0.9	11.5 ± 0.9	< 0.001
Platelet (/mm^3^)	318 ± 159	336 ± 98	0.012
Platelet, follow-up	513 ± 207	431 ± 155	< 0.001
CRP (mg/dL)	10.4 ± 6.8	8.8 ± 5.3	0.002
ESR (mm/h)	57 ± 23	74 ± 32	< 0.001
Total protein (g/dL)	6.5 ± 0.7	6.5 ± 0.6	0.368
Albumin (g/dL)	3.5 ± 0.4	3.8 ± 0.5	< 0.001
AST (IU/L)	77 ± 145	62 ± 75	0.128
ALT (IU/L)	111 ± 146	118 ± 174	0.625
ALT <40 IU, n (%)	131 (46.2)	156 (47.1)	0.654
ALT 41–99 IU, n (%)	63 (22.2)	68 (20.5)	0.684
ALT >100 IU, n (%)	90 (31.6)	107 (32.3)	0.863

Values are mean ± SD or n (%). Abbreviations as in Table 1

In laboratory indices, there were no significant differences in WBC count and differential, total protein, AST, or ALT values. However, there were significant differences in level of hemoglobin (10.8 g/dL vs. 11.5 g/dL, *P* < 0.001), platelet count at presentation (318,000/ mm^3^ vs. 336,000/ mm^3^, *P* = 0.012) and follow-up (513,000 vs. 431,000/mm^3^, *P* < 0.001), CRP (10.4 mg/dL vs. 8.8 mg/dL, *P* = 0.002), ESR (57 mm/h vs. 74/mm/h, *P* < 0*.*001), and albumin (3.5 g/dL vs. 3.8 g/dL, *P* < 0.001) (Table 2). The ESR estimation method was changed from the Wintrobe method to the automated analyzer method during the study period in both hospitals, which may have affected the values in this study.

Because some laboratory values can be affected by age, stage of disease including fever duration, and other factors, we analyzed the KD subgroup with a fever duration of 5 days or 6 days (*n* = 204), and the data are expressed in Table 3. Also, recent KD patients had shorter hospital stays, a higher proportion of incomplete KD, lower incidence of CALs, higher values of hemoglobin, albumin, and ESR, and lower CRP and follow-up platelet values compared to the patients with the same fever duration and age in the past KD patients (Table 3). Also, we analyzed the subgroup with complete KD (*n* = 387) and that with incomplete KD (*n* = 228), independently. The results of laboratory and clinical indices were nearly identical to those in the total KD group, although a few clinical parameters, such as CALs in the complete KD group, did not reach statistical significance (Tables 4 and 5). In the incomplete KD subgroup, there were no differences in the prevalence rates of each clinical diagnostic signs in both periods (data not shown).

**Table 3:** KD patients with fever duration of 5 or 6 days in the both groups (*n* = 204)

	2000–2004 (*n* = 100)	2010–2014 (*n* = 104)	*P*
Age (month)	31.1 ± 22	31 ± 20.1	0.978
Sex (M/F)	56/44	64/40	0.477
Fever duration (day)			
Preadmission	5.4 ± 0.5	5.3 ± 0.4	0.047
Total	7.1 ± 1.8	6.8 ± 1.5	0.104
Hospitalization (day)	8.6 ± 2.8	5.8 ± 1.8	< 0.001
Incomplete KD, n (%)	22 (22)	46 (44.2)	0.001
Repeated IVIG, n (%)	7 (7)	10 (9.6)	0.615
CALs, n (%)	31(31)	17 (16.3)	0.020
Eectasia	29 (29)	17 (16.3)	0.044
Aneurysm	2 (2)	0 (0)	0.239
WBC (/mm^3^)	15,500 ± 4800	14,800 ± 5100	0.318
Neutrophil (%)	65.7 ± 13.7	66.8 ± 15.0	0.572
Lymphocyte (%)	21.8 ± 11.7	23.2 ± 12.6	0.407
Hemoglobin (g/dL)	10.7 ± 0.8	11.4 ± 0.9	< 0.001
Platelet (x-10^3^/mm^3^)	323 ± 125	317 ± 78	0.038
Platelet, follow-up	532 ± 168	407 ± 120	< 0.001
CRP (mg/dL)	12.5 ± 7.1	9.3 ± 6.1	0.001
ESR (mm/h)	58 ± 24	77 ± 39	< 0.001
Total protein (g/dL)	6.4 ± 0.6	6.4 ± 0.6	0.848
Albumin (g/dL)	3.5 ± 0.4	3.7 ± 0.4	0.002
AST (IU/L)	59 ± 90	71 ± 110	0.397
ALT (IU/L)	102 ± 125	114 ± 177	0.570
ALT <40 IU, n (%)	46 (46)	43 (41.3)	0.573
ALT 41–99 IU, n (%)	23 (23)	33 (31.7)	0.209
ALT >100 IU, n (%)	31 (31)	28 (26.9)	0.540

Values are mean ± SD or n (%). Abbreviations as in Table 1

**Table 4:** Clinical and laboratory findings in complete KD in the 2000–2004 group and the 2010–2014 group (*n* = 387)

	2000–2004 (*n* = 210)	2010–2014 (*n* = 177)	*P*
Age (month)	30.9 ± 21.5	31 ± 18.9	0.938
Sex (M/F)	124/86	109/68	0.677
Fever (day)			
Preadmission	4.8 ± 1.8	4.3 ± 1.6	0.009
Total	6.9 ± 2.9	6.5 ± 2.3	0.392
Hospitalization (d)	8.5 ± 4.1	6.5 ± 2.8	< 0.001
Repeated IVIG, n (%)	24 (11.4)	22 (12.4)	0.754
CALs, n (%)	45 (21.4)	28 (15.8)	0.192
Ectasia	42 (20)	27 (15.3)	0.234
Aneurysm	3 (1.4)	1 (0.6)	0.629
WBC (/mm^3^)	15,100 ± 5100	15,000 ± 5200	0.880
Neutrophil (%)	64.5 ± 16.2	67.9 ± 14.2	0.031
Lymphocyte (%)	22.8 ± 12.8	22.1 ± 12.1	0.561
Hemoglobin (g/dL)	10.8 ± 1.2	11.5 ± 0.8	< 0.001
Platelet (/mm^3^)	315 ± 147	332 ± 89	0.122
Platelet, follow-up	501 ± 208	443 ± 161	< 0.001
CRP (mg/dL)	10.4 ± 6.8	9.5 ± 5.6	0.192
ESR (mm/h)	56 ± 24	74 ± 32	< 0.001
Total protein (g/dL)	6.5 ± 0.7	6.5 ± 0.6	0.656
Albumin (g/dL)	3.5 ± 0.4	3.8 ± 0.5	< 0.001
AST (IU/L)	89 ± 166	104 ± 156	0.338
ALT (IU/L)	127 ± 161	126 ± 149	0.928
ALT <40 IU, n (%)	89 (42.4)	75 (42.4)	0.918
ALT 40–100 IU, n (%)	45 (21.4)	36 (20.3)	0.900
ALT >100 IU, n (%)	76 (36.2)	66 (37.3)	0.750

Values are mean ± SD or n (%). Abbreviations as in Table 1

**Table 5:** Clinical and laboratory findings in incomplete KD in the 2000–2004 group and the 2010–2014 group (*n* = 228)

	2000–2004 (*n* = 74)	2010–2014 (*n* = 154)	*P*
Age (month)	29.3 ± 24.8	26.9 ± 22.0	0.456
Sex (M/F)	44/30	98/56	0.562
Fever (day)			
Preadmission	5.5 ± 2.8	4.2 ± 1.5	< 0.001
Total	7.9 ± 3.9	6.0 ± 1.6	< 0.001
Hospitalization (d)	8.6 ± 4.5	5.6 ± 1.7	< 0.001
Repeated IVIG, n (%)	6 (8.1)	7 (4.5)	0.358
CALs, n (%)	22 (29.7)	24 (15.6)	0.015
Ectasia	19 (25.7)	23 (14.9)	0.067
Aneurysm	3 (4.0)	1 (0.6)	0.102
WBC (/mm^3^)	15,500 ± 5000	14,800 ± 4900	0.291
Neutrophil (%)	60.5 ± 17.1	61.6 ± 14.8	0.599
Lymphocyte (%)	24.9 ± 13.7	27.7 ± 11.7	0.116
Hemoglobin (g/dL)	10.7 ± 0.8	11.4 ± 1.0	< 0.001
Platelet (/mm^3^)	324 ± 191	341 ± 108	0.010
Platelet, follow-up	550 ± 291	418 ± 146	< 0.001
CRP (mg/dL)	10.4 ± 6.8	8.0 ± 4.9	0.008
ESR (mm/h)	60 ± 23	70 ± 28	< 0.001
Total protein (g/dL)	6.4 ± 0.7	6.5 ± 0.6	0.703
Albumin (g/dL)	3.5 ± 0.4	3.9 ± 0.5	< 0.001
AST (IU/L)	43 ± 39	96 ± 297	0.030
ALT (IU/L)	65 ± 82	108 ± 174	0.021
ALT <40 IU, n (%)	42 (56.8)	81 (52.6)	0.888
ALT 40–100 IU, n (%)	18 (24.3)	32 (20.8)	0.616
ALT >100 IU, n (%)	14 (18.9)	41 (26.6)	0.241

Values are mean ± SD or n (%). Abbreviations as in Table 1

## Discussion

Since the first report of KD in the early 1960s in Japan, the disease has now appeared in over 60 countries around the world [10, 20]. Japan and Korea currently have the highest incidences of KD in the world; 264.8/100,000 in children aged <5 years in 2012 in Japan [21], and 194.7/10^5^ in 2014 in Korea [22]. KD has currently become one of the common diseases in Korea.

In the present study, we found that KD patients in Korea are increasing, but clinical and laboratory features might be changing to milder phenotypes over time. Nationwide epidemiological studies have indicated that the proportion of patients with incomplete KD was as high as 20% in 2007–2008 in Japan [23], and 42.2% of patients in 2009–2011 in Korea with no fatalities [15]. In this study, we confirmed this trend as well as in our previous studies [16, 17], the proportion of incomplete KD significantly increased from 26.4% in 2000–2004 to 46.2% in 2010–2014. In the present study, we found that incomplete KD patients had fewer cases of repeated IVIG treatment (5.7% vs. 11.9%, *P* = 0.015) and less severe values of some laboratory indices such as WBC differentials, CRP, albumin and ALT, but the rate of CALs in the incomplete KD patients was not different from that in complete KD patients (20.2% vs. 18.9%, *P* = 0.75) (Table 1). Other studies have also indicated that incomplete KD and complete KD patients had similar laboratory findings with similar incidences of CALs [24, 25]. These findings suggest that KD may appear as various phenotypes and immune responses against KD pathogen(s) and/or insults from the infection are similar in both KD patient groups, with individual variations such as involvement of ALT/ALT elevation and CALs. Thus, our results suggest that patients with incomplete KD may have milder clinical manifestations with less systemic inflammation reflected by some laboratory parameters compared to patients with complete KD. As for incomplete KD patients, Pediatricians in Korea have skillfully used other signs such as skin changes at the Bacille Calmette–Guérin inoculation site for infants or slit lamp examination to search the anterior uveitis for older children [26, 27], as well as laboratory findings.

The reduced risk of CALs in recent patients in this study could be attributable to early diagnosis and early IVIG in incomplete KD treatment as suggested in other studies [14, 15, 23]. Our KD patents in the 2010–2014 group also visited the hospital earlier (4.9 days vs. 4.2 days, *P* < 0.001) and subsequently they were treated with IVIG earlier than the patients in the past KD group. On the other hand, the complete and incomplete KD groups had the same preadmission fever duration (4.5 days vs. 4.6 days, *P* = 0.80), and incomplete KD patients received IVIG treatment later than complete KD patients (1.4 days vs. 1.1 days, *P* < 0.001), but there were no differences in CALs in the two groups. Therefore, other factors may also be associated with the low rate of CALs in recent years.

During a self-limited systemic inflammatory process in KD, there is a peak (mean 6th day after fever onset) in systemic inflammation, reflected in the laboratory parameters [9, 19]. We found that not only CALs but also some laboratory parameter values were less severe in the 2010–2014 KD patients than the 2000–2004 KD patients. In this series, CRP, albumin, hemoglobin, and platelet levels were significantly different in the two groups. Both groups of KD patients with the same fever duration (5 days or 6 days) and same mean age at presentation had identical results (Table 3). These findings were also observed when we analyzed patients with complete KD and those with incomplete KD separately in each time-period (Tables 4 and 5).

These laboratory indices are contained in the AHA diagnostic criteria for incomplete KD [1]. Albumin, hemoglobin, and CRP are very sensitive parameters that reflect systemic inflammation in acute KD, and they can vary significantly in daily examinations. Therefore, confirmation with the elevation of CRP, the reduction of albumin and hemoglobin, and the appearance of elevated transaminase and/or pyuria in daily examination may be helpful for early diagnosis in early-presented incomplete KD patients.

Platelet count may begin to increase after the peak of inflammation in the acute stage, suggesting that it has a role in the recovery reaction to KD [9, 19]. This was satisfied by all KD patients in this study, with each patient confirming platelet elevation during admission. In addition, we found that the platelet count and immunoglobulin G (IgG), IgM and IgA levels increased in the early convalescent stage of KD, and the extent of increase in these parameters correlated with each other [unpublished observation]. These findings suggest that platelets and immunoglobulins may be involved in the recovery from KD and the extent of increased the parameters in the subacute stage may reflect the extent of systemic inflammation. This characteristic of KD inflammation could be helpful for KD patient selection after IVIG therapy.

It has been reported that higher CRP, higher maximum platelet, lower albumin, or lower hemoglobin values are associated with IVIG-non responsiveness and the risk of CALs [28–31]. Our laboratory results provided evidence of changing epidemiology in KD in Korea, and this may be the first laboratory-proven study to demonstrate the results.

It has been reported that initially severe phenotypes of the diseases become milder over time in many infectious diseases, including scarlet fever, endocarditis, and pandemic influenza [11, 32, 33]. Also, these changes have been observed in infection-related immune-mediated diseases, such as acute rheumatic fever and APSGN [11–13]. Recent APSGN patients in Korea had a milder phenotype than patients in the past, although the incidence of the disease is markedly decreasing as well as in other developed countries [13]. Although the reason for changes in clinical features in KD is unknown, it could be attributable in part that KD pathogenic strain(s) or unknown herd immunity against the pathogens may have changed over time.

Because of early visit to hospital and changed phenotype of KD in Korea, application of the laboratory diagnostic criteria suggested by the AHA for incomplete KD patients with short fever duration can have some clinical obstacles, such as waiting for 1–2 days or more after admission [34]. Moreover, early IVIG treatment may affect the appearance of clinical diagnostic criteria of patients with potentially complete KD. These observations could explain, in part, why the number of patients with incomplete KD has increased in Korea.

This retrospective study may have some limitations. We analyzed diagnostic clinical signs through the initial description in medical records and applied diagnostic criteria regardless of the presence of CALs. Thus, additional signs that appeared during hospitalization could be omitted, and the number of incomplete KD patients could have been over-estimated. Although the number of subjects in this study was not small, this data may not be representative of the current situation in Korea. Finally, there might be some differences in patient care policies such as hospitalization and follow-up echocardiography, according to the time periods and different hospitals.

## Conclusions

Recently-admitted KD patients were more likely to present with incomplete KD and have a lower incidence of CALs, lower levels of CRP and platelet count, and higher levels of albumin and hemoglobin compared to similar patients a decade ago. These findings suggest that clinical features of KD became milder over time. Therefore, new diagnostic criteria for early IVIG treatment, including the laboratory criteria for patients that present early and confirmation after IVIG treatment for patient selection, may be needed in Korea in the near future.

## Abbreviations

ALT: Alanine aminotransferase
AST: Aspartate aminotransferase
CAL: Coronary artery lesion
CRP: C-reactive protein
ESR: Erythrocyte sedimentation rate
IVIG: Intravenous immunoglobulin
KD: Kawasaki disease
WBC: White blood cell
